# An Underwater 6-DoF Position and Orientation Estimation Method for Divers Based on the VideoPose5CH Model

**DOI:** 10.3390/s26041335

**Published:** 2026-02-19

**Authors:** Kaidong Wang, Yi Yang, Qingbo Wei, Xingqun Zhou, Zhiqiang Hu, Quan Zheng

**Affiliations:** 1 State Key Laboratory of Robotics and Intelligent Systems, Shenyang Institute of Automation, Chinese Academy of Sciences, Shenyang 110016, China; wangkaidong@sia.cn (K.W.);; 2 University of Chinese Academy of Sciences, Beijing 100049, China; 3 School of Electronic and Information Engineering, Harbin Institute of Technology, Shenzhen 150001, China

**Keywords:** underwater human–robot collaboration, underwater vision, 6-DoF position and orientation estimation, human pose estimation, data augmentation

## Abstract

Accurate perception of a diver’s position and orientation by Autonomous Underwater Vehicles (AUVs) is essential for effective human–robot collaboration in underwater environments. However, conventional position and orientation estimation methods that combine deep learning with Perspective-n-Point (PnP) algorithms are primarily designed for rigid objects. In contrast, divers exhibit highly variable postures underwater, with no fixed configuration. To address this limitation, this paper proposes a framework for estimating the six-degree-of-freedom (6-DoF) position and the orientation of a diver. In addition, a novel network architecture, termed “VideoPose5CH,” is proposed. In the proposed framework, temporal sequences of 2D joint coordinates are provided to VideoPose5CH, which then outputs the 3D joint coordinates of the current frame as well as the corresponding refined 2D joint locations. Subsequently, the diver’s 6-DoF position and orientation relative to the camera are further recovered via a PnP algorithm. To mitigate the scarcity of underwater 3D human pose datasets, a land-based 3D human pose dataset augmentation strategy tailored to underwater conditions is further proposed. With this strategy, diver pose estimation accuracy is improved and the robustness of the proposed method across diverse scenarios is enhanced. Experimental results demonstrate that the proposed method can stably estimate the 6-DoF position and orientation of the diver within a distance range of 2.643 m to 11.477 m. The average position errors along the three axes are 7.33 cm, 4.04 cm, and 27.15 cm, respectively, while the average orientation errors are 6.96°, 8.47°, and 2.62°.

## 1. Introduction

In recent years, Autonomous Underwater Vehicles (AUVs) have been increasingly deployed in a wide range of underwater operations, including marine resource exploration, seabed mapping, and military missions [1]. Despite these advances, human divers remain indispensable in complex scenarios such as shipwreck detection, the inspection of damaged infrastructure, and underwater search and rescue [2,3,4]. Collaborative operations between divers and AUVs have been shown to significantly improve task efficiency, reduce the physical workload imposed on divers [5], and enhance operational safety [6]. A fundamental requirement for such human–robot collaboration is the underwater robot’s accurate perception of the diver’s position and orientation. However, reliable perception of the diver’s position and orientation in underwater environments remains highly challenging. Severe light attenuation in underwater environments renders conventional terrestrial localization techniques, such as light detection and ranging (LiDAR) and infrared-based devices, ineffective. Meanwhile, sonar-based sensing, which is widely adopted underwater, is often bulky, expensive, and incapable of providing fine-grained human orientation information [7]. In contrast, monocular cameras offer advantages such as low cost, compact size, light weight, and ease of integration [8], making them highly advantageous for perception tasks.

Despite the significant progress achieved by vision-based pose estimation methods, accurate estimation of the position and orientation of divers in underwater environments remains highly challenging. First, regarding visual perception of the human body, most existing studies have primarily focused on human skeletal pose estimation [9,10,11,12], with relatively few addressing methods for perceiving the 6-DoF position and orientation of the human body. Second, among vision-based 6-DoF position and orientation estimation approaches, a widely adopted, highly accurate solution combines deep learning with the Perspective-n-Point (PnP) algorithm [8,13,14,15]. Specifically, a neural network is first employed to extract the 2D pixel coordinates of predefined keypoints from an image. Given the prior knowledge of the corresponding 3D keypoint coordinates of the target object, the object’s position and orientation with respect to the camera coordinate system are then recovered by solving the PnP problem using these 2D–3D correspondences. However, such methods are generally designed for rigid objects with fixed geometries. In the case of divers, continuous motion and deformation underwater lead to constantly changing body shapes, rendering conventional deep learning–PnP-based pose estimation methods inapplicable. Third, acquiring underwater 3D human pose datasets is inherently difficult. In terrestrial environments, the 3D coordinates of human keypoints are typically obtained using motion capture (MoCap) systems; however, deploying such systems underwater is technically infeasible and prohibitively expensive. Alternatively, constructing underwater 3D human pose datasets using stereo cameras or similar setups is further constrained by factors such as bubbles interference, the limited availability of real underwater images or videos, and the high cost of manual annotation. Consequently, effective approaches for building underwater 3D human pose datasets are lacking, which severely limits the applicability of supervised learning approaches.

To address the above challenges, this paper proposes a monocular 6-DoF position and orientation estimation method for divers and introduces a novel network, termed VideoPose5CH. Specifically, the proposed approach employs the VideoPose5CH network to predict the 2D and 3D coordinates of selected diver’s keypoints, and then estimates the diver’s 6-DoF position and orientation with respect to the camera coordinate system by using a Random Sample Consensus (RANSAC)-based PnP method. To facilitate the training of VideoPose5CH for underwater diver scenarios, a data augmentation strategy is designed, which leverages terrestrial datasets to construct an underwater 3D human pose dataset. The contributions of this paper are summarized as follows.

(1) A Human Pose Estimation Model, VideoPose5CH, is Proposed for the Position and Orientation Estimation Task. This model is based on a causal dilated convolutional neural network. The model takes the 2D keypoint coordinates produced by a 2D detector from the current frame and the preceding 242 frames as input, and outputs the 3D keypoint coordinates of the current frame as well as refined 2D keypoints. The accurate 2D–3D keypoint estimates generated by the model could facilitate the subsequent robust estimation of 6-DoF position and orientation using the RANSAC-based PnP algorithm.
(2) A 6-DoF Diver’s Position and Orientation Estimation Framework is Established Without Explicit Underwater Position and Orientation Annotations. A novel 6-DoF diver’s position and orientation estimation framework is proposed, which does not require explicit position and orientation labels in underwater environments. The framework decomposes the estimation into two stages: (i) estimating 2D joint locations of the diver in the image plane, and (ii) the corresponding 3D joint coordinates are estimated while refining the 2D joint locations based on the initial 2D predictions. The dataset for the first stage can be obtained through manual annotation, whereas the second-stage training data can be constructed by augmenting terrestrial datasets. This method effectively avoids the difficulty of acquiring the diver’s position and orientation annotations in underwater scenarios.
3) An Underwater-Oriented Data Augmentation Strategy is Proposed for the Construction of 3D Human Pose Datasets. A novel data augmentation method is developed to transfer terrestrial 3D human pose datasets to underwater scenarios. Since underwater diver’s orientations are generally more complex than those in terrestrial settings, directly regressing 3D coordinates from 2D pixels using only land-based datasets may lead to substantial errors. To mitigate this problem, an augmentation strategy is proposed that combines depth and angle augmentations, significantly enhancing the generalization ability of the VideoPose5CH model.

## 2. Related Work

This section reviews the related studies on human pose estimation, including 2D human pose estimation, 3D human pose estimation, and the major application scenarios of human pose estimation.

### 2.1. 2D Human Pose Estimation

2D human pose estimation has long been a fundamental problem in computer vision. Its primary objective is to localize human body keypoints (e.g., elbows, knees, and wrists) by predicting their pixel coordinates from 2D images or videos sequences.

Based on the form of model outputs, 2D human pose estimation methods can be broadly categorized into direct regression–based approaches and heatmap-based approaches. Direct regression–based methods explicitly predict the 2D coordinates of human body keypoints. DeepPose [16] was one of the earliest works to apply deep neural networks to keypoint coordinate regression, employing a cascade of Deep Neural Network (DNN)-based pose regressors. In its first stage, the full image is utilized to produce coarse keypoint estimates, while in subsequent stages, high-resolution image patches are cropped around the predicted joints to progressively refine the keypoint locations. To address the limitation of feed-forward networks in modeling output-space dependencies, Carreira et al. [17] proposed the Iterative Error Feedback (IEF) framework. In this approach, a top-down feedback mechanism is introduced to iteratively refine pose predictions, thereby explicitly modeling structured dependencies among keypoints in the output space and significantly improving regression accuracy.

Heatmap-based approaches use heatmaps as network outputs, where each 2D joint is modeled as a spatial probability distribution, and joint coordinates are obtained by locating the maximum responses. In general, these methods achieve high localization accuracy while providing rich spatial context and dense supervisory signals. Convolutional Pose Machines (CPM), proposed by Wei et al. [18], employ a multi-stage cascaded convolutional architecture to progressively refine joint heatmap predictions, in which long-range spatial dependencies among body parts are implicitly captured through expanded receptive fields. The Cascaded Pyramid Network (CPN) [19] was designed to address the localization of so-called “hard keypoints” in human pose estimation. It consists of two components, namely GlobalNet and RefineNet: GlobalNet primarily localizes “easy keypoints,” whereas RefineNet explicitly refines “hard keypoints” that are occluded or require global contextual reasoning, thereby improving robustness in complex multi-person scenarios. The High-Resolution Network (HRNet), proposed by Sun et al. [10], introduced a novel architectural paradigm that maintains high-resolution feature representations throughout the inference process, enabling more accurate heatmap prediction. Despite the substantial performance gains achieved by Convolutional Neural Network (CNN)-based methods, their limited interpretability makes it difficult to intuitively understand how spatial dependencies among keypoints are modeled. To alleviate this issue, inspired by the ability of Transformer-based attention mechanisms to capture long-range dependencies and explicitly model interactions among keypoints, Yang et al. [20] proposed the TransPose network, which preserves high-precision heatmap outputs while significantly enhancing the interpretability of keypoint dependency modeling.

For multi-person 2D human pose estimation, existing methods are generally divided into two paradigms: top-down and bottom-up approaches. Top-down methods first localize human bounding boxes using a person detector, after which single-person pose estimation is performed independently within each detected region. Representative examples include HRNet [10] and CPN [19]. In contrast, bottom-up methods initially detect all candidate body keypoints over the entire image and subsequently associate them with individual persons. Among these, OpenPose, proposed by Cao et al. [9], is one of the most representative approaches. It introduces Part Affinity Fields (PAFs) to encode the spatial and orientation relationships between human limbs, enabling person instances to be assembled via bipartite matching. Notably, OpenPose was the first framework to robustly estimate multi-person poses in images or videos while maintaining real-time performance.

### 2.2. 3D Human Pose Estimation

3D human pose estimation aims to recover the 3D coordinates of human body joints from images or videos. Due to the inherent depth ambiguity in monocular settings, the mapping from 2D observations to 3D pose configurations is fundamentally under-constrained, as a single 2D pose may correspond to multiple plausible 3D poses. Consequently, monocular 3D human pose estimation has become both the most extensively studied and one of the most challenging problems in this field [21]. To mitigate this intrinsic ambiguity, numerous methods have been proposed, which can be broadly categorized into two classes: direct regression–based approaches and 2D-to-3D lifting–based approaches.

Direct regression methods estimate the 3D joint coordinates end-to-end from RGB images, without explicitly predicting intermediate 2D keypoint locations. The work by Li et al. [22] was among the first to directly regress 3D joint coordinates from monocular images using deep neural networks. To address the lack of in-the-wild datasets with 3D annotations, Zhou et al. [23] proposed a weakly supervised 3D human pose estimation framework that combines 2D and 3D supervision: it leverages 2D annotations from in-the-wild images to provide weak supervisory signals for 3D pose estimation. Experimental results demonstrate that this method generalizes well on the MPI-INF-3DHP dataset [24], which includes 3D pose annotations captured in outdoor scenarios.

2D-to-3D lifting methods first employ a well-established 2D pose estimator to extract 2D keypoint coordinates from images, which are then fed into a lifting network to regress the corresponding 3D joint coordinates. Benefiting from the strong performance of modern 2D pose detectors, 2D-to-3D lifting pipelines often outperform direct regression–based approaches [25]. An early representative work is SimpleBaseline3D, proposed by Martinez et al. [11], which regresses 3D joint positions from single-frame 2D joint detections using a simple yet effective fully connected residual network. Beyond single-frame inputs, video sequences provide rich temporal cues that significantly improve the accuracy and robustness of 3D pose prediction. Pavllo et al. [12] proposed VideoPose3D, which leverages dilated temporal convolutions to enlarge the temporal receptive field and effectively capture long-term dependencies in sequential data. However, temporal convolution–based methods are inherently constrained by a finite temporal window. To more effectively model global correlations in long video sequences, Zheng et al. [25] proposed PoseFormer, the first 3D human pose estimation framework based on a purely Transformer architecture. PoseFormer introduces Transformer modules in both the spatial and temporal domains to explicitly model inter-joint spatial dependencies and inter-frame temporal dependencies, respectively. This design enhances spatiotemporal feature representation while maintaining relatively low memory consumption for long-sequence inputs.

### 2.3. Application of Human Pose Estimation

From an application perspective, existing research on human pose estimation has primarily focused on domains such as healthcare, sports training and analysis, human–computer interaction, and security surveillance. In the healthcare domain, Chen et al. [26] proposed a lightweight regression model based on collaborative learning. By integrating 2D and 3D pose assessment with pose tracking techniques, their method enables real-time estimation of 3D poses of construction workers from monocular videos, which can be applied to ergonomic risk assessment of work-related musculoskeletal disorders. Liu et al. [27] employed human pose estimation for fall risk detection in older adults. Skeletal keypoints were first extracted using OpenPose, after which interpretable physical features, such as body proportions, acceleration, and deflection angles, were constructed. High-accuracy fall event recognition was subsequently achieved using a k-nearest neighbors (KNN) classifier. In the field of sports training and performance analysis, Wu et al. [28] addressed the limitations of existing pose estimation models in terms of keypoint localization accuracy and real-time performance under fast-motion scenarios, such as badminton. By introducing an Efficient Local Attention (ELA) mechanism into the YOLOv8-Pose architecture and constructing a dedicated badminton pose dataset, both localization accuracy and real-time performance were significantly improved. For human–computer interaction applications, Cheng et al. [29] addressed the limitations of depth-camera-based interaction frameworks, including restricted operating distance and reliance on specialized hardware, by proposing an interaction method that combines human pose estimation with motion intention recognition. In their approach, 3D joint information is extracted from RGB images and fed into an LSTM-based intention recognition network to map multi-frame joint sequences to human action intentions. Compared with depth-camera-based methods, this approach supports longer interaction distances and achieves stable and effective performance under varying distances and lighting conditions. In the security surveillance domain, Cormier et al. [30] addressed challenges such as top-down viewpoints, severe occlusions, and stringent real-time requirements encountered in the automatic recognition of violent or public safety–threatening behaviors. They developed a lightweight processing framework that integrates human detection, pedestrian re-identification and tracking, and top-down pose estimation, thereby significantly improving pose trajectory continuity and behavior recognition reliability in complex surveillance environments.

In summary, most application-oriented studies of human pose estimation focus on recognizing human body configurations and actions. In this work, we extend pose estimation to underwater settings by estimating the 6-DoF position and orientation of divers.

## 3. Methodology

### 3.1. 6-DoF Position and Orientation Estimation for Divers Based on VideoPose5CH Model

**Figure 1:**
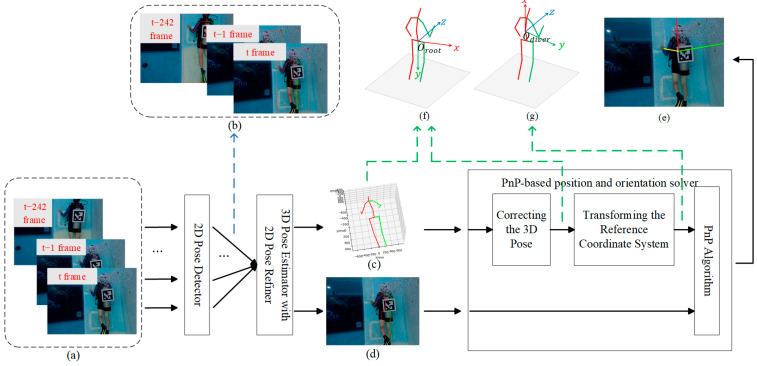
Overall framework of the proposed diver’s 6-DoF position and orientation estimation method. (**a**) Input sequence (current frame and previous 242 frames). (**b**) 2D pose sequence from the 2D pose detector. (**c**) Current-frame 3D pose in the root-joint coordinate system (VideoPose5CH output). (**d**) Refined current-frame 2D pose. (**e**) Estimated current-frame position and orientation w.r.t. the camera coordinate system, shown as a 3D Cartesian frame, where the red, green, and yellow axes correspond to the x-, y-, and z-axis directions, respectively. (**f**) Root-joint coordinate system. (**g**) Body-fixed coordinate system. Solid lines denote data flow. the blue dashed arrow specifies the data carried along the flow, while green dashed arrows denote the coordinate system in which the 3D pose is defined.

The proposed method for estimating the 6-DoF position and orientation of divers based on the VideoPose5CH model is presented in this section. An overview of the framework is illustrated in Figure 1. The proposed approach consists of three main components: a 2D pose detector, a 3D pose estimator with an integrated 2D pose refinement module, and a PnP-based position and orientation solver. The 2D pose detector is first employed to estimate the diver’s 2D pose from a single image frame. Subsequently, the 3D pose estimator with 2D pose refinement takes the sequence of 2D poses from the current frame and the preceding 242 frames as input, and regresses the 3D pose of the current frame in the root-joint coordinate system. Meanwhile, a refined 2D pose for the current frame is also produced. Given the estimated 3D pose and its corresponding refined 2D pose, the PnP-based position and orientation solver computes the diver’s 6-DoF position and orientation with respect to the camera coordinate system using the RANSAC-based Perspective-n-Point (PnP) algorithm. In the following subsections, we first introduce the construction of the root-joint coordinate system and the body-fixed coordinate system, which helps clarify the coordinate frames used for the 3D joint positions in the subsequent sections. Then, the three main modules illustrated in Figure 1 are described in detail.

#### 3.1.1. Construction of the Root-Joint Coordinate System and the Body-Fixed Coordinate System

In monocular image-based 3D human pose estimation, the absence of depth information leads to scale ambiguity. To mitigate this issue, the diver’s 3D pose estimated by the 3D pose estimator with the 2D pose refiner is represented in the root-joint coordinate system. By expressing each joint using its coordinates relative to the root joint, the scale variation in the 3D pose caused by factors such as camera-to-diver distance and inter-subject body size can be effectively reduced [11,22,31].

**Figure 2:**
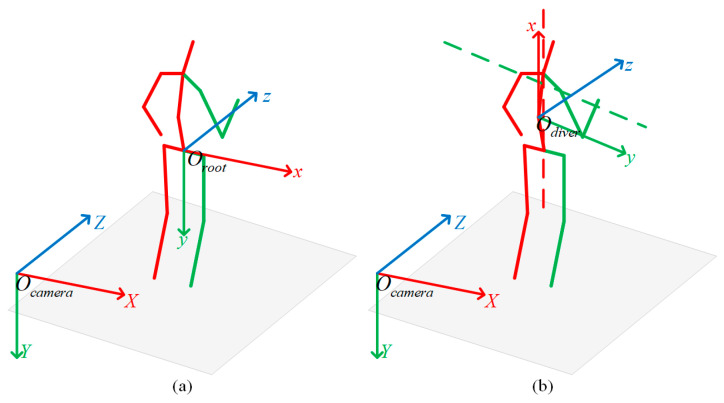
Root-joint coordinate system and body-fixed coordinate system. (**a**) Root-joint coordinate system, where $O_{camera}$ and $O_{root}$ denote the origins of the camera coordinate system and the root-joint coordinate system, respectively. (**b**) Body-fixed coordinate system, where $O_{diver}$ denotes the origin of the body-fixed coordinate system; the red dashed line indicates the segment connecting the hip joint and the thorax, and the green dashed line indicates the segment connecting the left and right shoulders. For the human skeleton shown in the figure, the red and green solid lines indicate the left and right sides of the skeleton, respectively.

As shown in Figure 2a, the hip joint is selected as the root joint and defined as the origin of the root-joint coordinate system ($O_{root}$). The three axes of the root-joint coordinate system are aligned with those of the camera coordinate system.

To describe the position and orientation of the diver relative to the camera coordinate system, a body-fixed coordinate system is established on the diver using torso-related joint keypoints, as illustrated in Figure 2b. The origin of this coordinate system is denoted as $O_{diver}$. In this study, the diver’s position and orientation are represented by the rigid transformation between the body-fixed coordinate system and the camera coordinate system. The body-fixed coordinate system is defined as follows:

(a) The spine joint is chosen as the origin of the body-fixed coordinate system;
(b) The positive x-axis is defined as the direction from the hip joint toward the thorax;
(c) A pseudo y-axis is defined as the direction from the left shoulder to the right shoulder;
(d) The positive z-axis is set to the direction of the cross product between the unit x-axis vector and the unit pseudo-y vector;
(e) The positive y-axis is set to the direction of the cross product between the unit z-axis vector and the unit x-axis vector.

It should be noted that a human diver is not a strictly rigid object. Therefore, the proposed torso-based body-fixed coordinate system is defined using torso-related keypoints (i.e., the hips, spine, thorax, and shoulders) to approximate the diver’s overall motion. Although the torso is more stable than the limbs, it is not strictly rigid during active swimming. Such residual torso deformation may induce slight variations in the constructed coordinate axes as well as the corresponding 3D keypoint configuration. In the proposed framework, the PnP solver is applied in a frame-by-frame manner and does not rely on a fixed rigid torso template; instead, the current-frame rigid transformation between the camera coordinate system and the body-fixed coordinate system is estimated. Consequently, the estimated position and orientation can be interpreted as the best rigid approximation of the diver’s torso motion in that frame, which remains of practical significance for downstream applications such as diver tracking as well as underwater human–robot collaboration.

#### 3.1.2. 2D Pose Detector

The 2D pose detector is used to obtain the 2D pose of a single diver in each image frame. Specifically, for a single-frame image $I_{t}\in {\mathbb{R}}^{W\times H\times 3}$ (where W and H denote the image width and height, respectively, and t is the frame index), the 2D pose detector outputs the diver’s 2D pose $P_{2D}^{\left(t\right)}={\left\{\left(u_{i}^{t},v_{i}^{t}\right)\right\}}_{i=0}^{N}$. Here, $\left(u_{i}^{t},v_{i}^{t}\right)$ denotes the pixel coordinate of the i-th joint in the t-th frame, and N is the number of joints used in this study.

A substantial body of mature research has been established in the field of 2D human pose estimation. Representative methods such as OpenPose [9], CPN [19], and HRNet [10] have demonstrated strong performance in terms of both real-time efficiency and detection accuracy. Building upon these advances, HRNet is adopted as the 2D pose detector in the proposed approach.

In heatmap-based 2D human pose estimation, HRNet [10] introduces a novel network architecture. Unlike conventional approaches that typically employ a cascaded high-to-low-resolution design—where low-resolution feature representations are first extracted by an encoder and then upsampled by a decoder to generate joint heatmaps—HRNet maintains high-resolution representations throughout the entire network. The core idea of HRNet is to run multiple subnetworks at different resolutions in parallel rather than connecting them sequentially. Meanwhile, repeated multi-scale feature fusion is performed through multiple “exchange units,” enabling each branch to continuously receive information from other resolution branches, and such fusion operations are performed eight times throughout the network. Benefiting from this design, HRNet produces more accurate joint heatmaps, thereby substantially improving joint localization accuracy. As a result, HRNet achieves state-of-the-art performance on benchmark datasets such as the COCO keypoint detection dataset [32] and the MPII human pose dataset [33].

In this study, HRNet is adopted as the 2D pose detector, which serves as a high-quality and reliable input for the subsequent 3D pose estimator with 2D pose refiner.

#### 3.1.3. 3D Pose Estimator with 2D Pose Refiner

A 3D pose estimator with a 2D pose refiner is designed to recover the 3D joint coordinates of the current frame, expressed in the root-joint coordinate system, from the 2D joint coordinate sequence of the current frame and the preceding 242 frames, while simultaneously outputting refined 2D joint coordinates. A 2D pose sequence of length 243 frames is used as input to fully exploit temporal information, thereby yielding more accurate and stable 3D pose estimation results. Although the initial 2D joint coordinates are already provided by the 2D pose detector, the 3D pose estimation with 2D pose refinement module is still introduced to re-estimate a consistent set of 2D poses. This is done to align the joint definitions between the 2D and 3D poses and to avoid additional errors in the subsequent PnP-based solution of the 6-DoF position and orientation, which could be caused by inconsistent joint sets between the datasets used to train the 2D pose detector and those used to train the 3D pose estimator with the 2D pose refiner.

**Figure 3:**
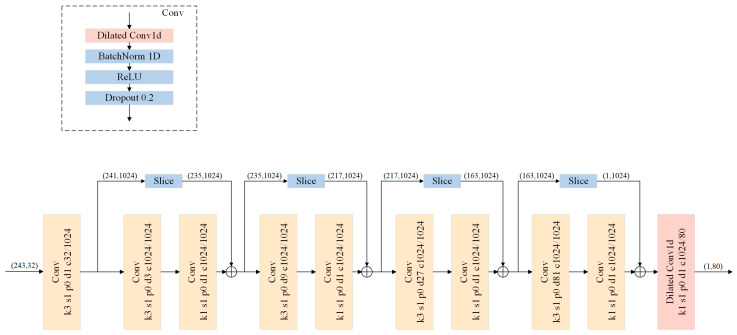
Architecture of the VideoPose5CH network. In the convolution modules, k3, s1, p0, d1, and c32/1024 indicate the kernel size, stride, padding, dilation factor, and input/output channels, respectively (3, 1, 0, 1, and 32/1024). To enable valid convolution, the residual (retaining the data on the right side) is sliced to match the shape of the subsequent tensor.

Accordingly, inspired by VideoPose3D [12], a high-performance model for 3D human pose estimation, the VideoPose5CH model is proposed. From a model architecture perspective, the VideoPose5CH model is specifically designed to better support the downstream position and orientation estimation task. Unlike the baseline VideoPose3D model, which outputs only the 3D joint coordinates of the middle frame, VideoPose5CH modifies the output channels of the final convolutional layer and employs causal convolutions to simultaneously predict the 3D joint coordinates and the corresponding refined 2D joint coordinates of the current frame. To supervise the optimization of the 3D and 2D outputs, separate loss functions are designed for the two tasks, which will be introduced later. The input to the model is a tensor of size 243×(16×2), formed by concatenating the 2D coordinates of 16 joints across 243 frames. The output tensor has a size of $1\times \left[16\times \left(3+2\right)\right]$, which comprises the 3D coordinates of the 16 joints in the current frame expressed in the root-joint coordinate system, together with the corresponding refined 2D coordinates. VideoPose5CH is designed as a fully convolutional neural network based on causal dilated convolutions. By introducing a dilation factor, dilated convolution exponentially enlarges the receptive field without increasing the number of parameters, which is particularly beneficial for modeling long-range temporal dependencies. The overall architecture of VideoPose5CH is illustrated in Figure 3 and consists of an input layer, a series of intermediate layers, and an output layer. The input layer includes a convolutional layer followed by Batch Normalization, a ReLU activation, and a Dropout layer, whereas the output layer contains only a single convolutional layer. The intermediate stage comprises four residual blocks with skip connections between blocks. Each residual block contains two convolutional layers, each followed by Batch Normalization, ReLU, and Dropout. Specifically, the first convolution in each block is a dilated convolution with a kernel size of 3, where the dilation factor increases with the block depth; the second convolution is the standard convolution.

During model training, the input data consist of the 2D joint coordinates obtained by applying the 2D pose detector used in this study to all video frames in the 3D human pose datasets. The supervision signals comprise two components: 3D pose labels and refined 2D pose labels. The 3D pose labels are formed by the 3D joint coordinates expressed in the root-joint coordinate system, which are computed by subtracting the root joint’s 3D coordinate from each joint’s 3D coordinate in the camera coordinate system. The refined 2D pose labels are constructed by projecting the joints’ 3D coordinates in the camera coordinate system onto the image plane using the camera projection model. The 2D coordinates obtained through geometric projection of the 3D joint coordinates are adopted as refined 2D labels for two main reasons. First, mainstream 3D human pose datasets are typically collected using motion capture (MoCap) systems, which provide highly accurate and temporally stable 3D annotations with minimal jitter. Second, given the joints’ 3D coordinates in the camera coordinate system and the corresponding camera parameters, the 2D coordinates computed via the deterministic geometric projection relationship are likewise accurate and temporally stable. Consequently, these projected 2D coordinates satisfy the requirements for refined 2D pose supervision in the proposed framework.

The loss function of VideoPose5CH is designed to optimize the accuracy of both the estimated 3D pose and the refined 2D pose. It consists of a 3D pose loss and a refined 2D pose loss. Specifically, the 3D pose loss is defined as the mean per-joint position error (MPJPE), while the refined 2D pose loss is computed using the Smooth L1 loss. These losses are defined as follows:

(1) $$L_{3D}=\frac{1}{N}\sum_{i=0}^{N-1}{\left\|\hat{P_{i}}-P_{i}\right\|}_{2}$$

(2) $$L_{2D}=\frac{1}{2N}\sum_{i=0}^{N-1}\left\{\begin{array}{ll} \frac{1}{2\beta }{\left\|\hat{p_{i}}-p_{i}\right\|}_{2}^{2}, & {\left\|\hat{p_{i}}-p_{i}\right\|}_{1}<\beta \\ {\left\|\hat{p_{i}}-p_{i}\right\|}_{1}-\frac{\beta }{2}, & {\left\|\hat{p_{i}}-p_{i}\right\|}_{1}\geq \beta \end{array}\right.$$

where N denotes the number of joints; $\hat{P_{i}}$ and $P_{i}$ are the 3D coordinates of the i-th joint predicted by VideoPose5CH and the corresponding ground truth, respectively; $\hat{p_{i}}$ and $p_{i}$ are the refined 2D coordinates of the i-th joint predicted by VideoPose5CH and the corresponding ground truth, respectively. ${\left\|\cdot \right\|}_{1}$ and ${\left\|\cdot \right\|}_{2}$ denote $L_{1}$ and $L_{2}$ norms, respectively. And β is the transition parameter of the Smooth L1 loss, which determines the point at which the loss function transitions from the L2 loss to the L1 loss, thereby balancing precise fitting for small errors and robustness to outliers. As a result, the total loss is given by

(3) $$L=\lambda L_{3D}+(1-\lambda )L_{2D},$$

where λ is a weighting parameter that balances the 3D pose loss and 2D pose loss.

#### 3.1.4. PnP-Based Position and Orientation Solver

The PnP-based solver first corrects the 3D pose output from the previous module and transforms the joint 3D coordinates from the root-joint coordinate system to the body-fixed coordinate system. Subsequently, the position and orientation of the diver’s body-fixed coordinate system with respect to the camera coordinate system are estimated using a RANSAC-based PnP algorithm, based on the correspondences between the 2D joint coordinates in the image plane and the associated 3D joint coordinates expressed in the body-fixed coordinate system.

1. Correcting the 3D Pose

To further reduce the influence of variations in the distance between the diver and the camera on the scale of the 3D pose, the 3D pose output by the VideoPose5CH model is corrected using the actual lengths of the diver’s limbs. First, the mean scaling ratio s between the limb lengths estimated from the model output and their corresponding actual lengths is computed. Let $\hat{l_{j}}$ and $l_{j}$ denote the estimated length and the actual length of the j-th limb, respectively, where the limbs include eight segments such as the left upper arm, left forearm, right thigh, and right shank. The scaling ratio s is computed as follows:

(4) $$s=\frac{1}{J}\sum_{j=0}^{J-1}\frac{l_{j}}{\hat{l_{j}}},J=8$$

where J is the number of limbs used in this study, i.e., J=8. The computed scaling ratio is then applied to uniformly scale the 3D pose output by VideoPose5CH to obtain the corrected pose:

(5) $${\hat{P_{i}}}^{corr}=s\cdot \hat{P_{i}}$$

where $\hat{P_{i}}$ denotes the original 3D pose of the i-th joint predicted by the model, and ${\hat{P_{i}}}^{corr}$ denotes the corrected 3D pose.

2. Transforming the Reference Coordinate System of Joint 3D Coordinates

To apply the PnP algorithm to estimate the diver’s position and orientation with respect to the camera coordinate system—i.e., to solve for the position and orientation of the body-fixed coordinate system relative to the camera coordinate system—the 3D joint coordinates must be expressed in the body-fixed coordinate system. However, the 3D joint coordinates output by VideoPose5CH, which are subsequently corrected based on the diver’s limb length, are expressed in the root-joint coordinate system. Therefore, a coordinate transformation is required to convert the joint 3D coordinates from the root-joint coordinate system to the body-fixed coordinate system.

For this purpose, the position and orientation of the root-joint coordinate system relative to the body-fixed coordinate system are first computed according to the body-fixed coordinate system construction described in Section 3.1.1. Based on a subset of joint coordinates provided by the 3D coordinate correction module (see Table 1), specially PHipRoot=03×1, the unit direction vectors of the body-fixed coordinate axes expressed in the root-joint coordinate system can be obtained: the x-axis direction vector is given in Equation (6), the pseudo y-axis direction vector in Equation (7), the z-axis direction vector in Equation (8), and the y-axis direction vector in Equation (9). Accordingly, the rotation matrix of the body-fixed coordinate system with respect to the root-joint coordinate system can be constructed as in Equation (10). By taking the matrix inverse, the rotation matrix of the root-joint coordinate system with respect to the body-fixed coordinate system is obtained, as shown in Equation (11). Finally, the position vector of the root-joint coordinate system with respect to the body-fixed coordinate system, i.e., the coordinates of the origin of the root-joint coordinate system expressed in the body-fixed coordinate system, is given in Equation (12), where RootO denotes the symbol used to represent the origin of the root coordinate system.

(6) XDiverRoot=PThoraxRoot−PHipRootPThoraxRoot−PHipRoot2

(7) YDiver∗Root=PRShoulderRoot−PLShoulderRootPRShoulderRoot−PLShoulderRoot2

(8) ZDiverRoot=XDiverRoot×YDiver∗Root

(9) YDiverRoot=ZDiverRoot×XDiverRoot

(10) RDiverRoot=[XDiverRootYDiverRootZDiverRoot]

(11) RRootDiver=R−1DiverRoot

(12) PRootODiver=−PSpineRoot

After obtaining the position and orientation of the root-joint coordinate system relative to the body-fixed coordinate system, the 3D joint coordinates can be transformed from the root-joint coordinate system to the body-fixed coordinate system as follows:

(13) Pi^Diver=PRootODiver+RRootRootDiverPi^

where Pi^Root denotes the 3D coordinate of the i-th joint in the root-joint coordinate system (i.e., ${\hat{P_{i}}}^{corr}$ in Equation (5)), and Pi^Diver represents the 3D coordinate of the i-th joint in the body-fixed coordinate system.

Given the 3D joint coordinates in the body-fixed coordinate system and the corresponding 2D joint coordinates in the image, the PnP algorithm is then employed to estimate the position and orientation of the body-fixed coordinate system relative to the camera coordinate system. The PnP algorithm estimates the 6-DoF position and orientation of an object by minimizing the reprojection error [7]. The mathematical model is expressed as

(14) ER^,t^=∑ipi^−πR^Pi^Diver+t^2

where $\hat{R}$ is the rotation matrix of the body-fixed coordinate system relative to the camera coordinate system, $\hat{t}$ is the position of the body-fixed coordinate system relative to the camera coordinate system, Pi^Diver denotes the 3D coordinates of joints in the body-fixed coordinate system, π(⋅) is the projection function that maps Pi^Diver onto the image plane via the camera model, and $p_{i}$ denotes the observed 2D projections of the joints in the image.

### 3.2. Terrestrial 3D Human Pose Dataset Augmentation Tailored for Underwater Environments

Due to the unique characteristics of underwater environments, motion-capture systems are difficult to deploy, and methods that obtain 3D joint coordinates based on stereo vision are highly susceptible to occlusions. Consequently, it is extremely challenging to acquire 3D human pose datasets for underwater divers. To address this issue, this section proposes a practical solution for constructing an underwater 3D pose dataset by augmenting terrestrial datasets, targeting diver’s 3D pose estimation methods that lift 2D poses to 3D poses. The proposed approach includes two augmentation strategies: depth augmentation and angle augmentation. The following subsections provide a detailed introduction to these two augmentation methods, and then describe how they are combined with the terrestrial 3D human pose dataset, the Human3.6M Dataset [34], to generate augmented training data for the proposed VideoPose5CH model.

#### 3.2.1. Depth Augmentation

Depth augmentation improves the generalization ability of the VideoPose5CH model across different depths by modifying the coordinates of the human joints in the camera coordinate system.

**Figure 4:**
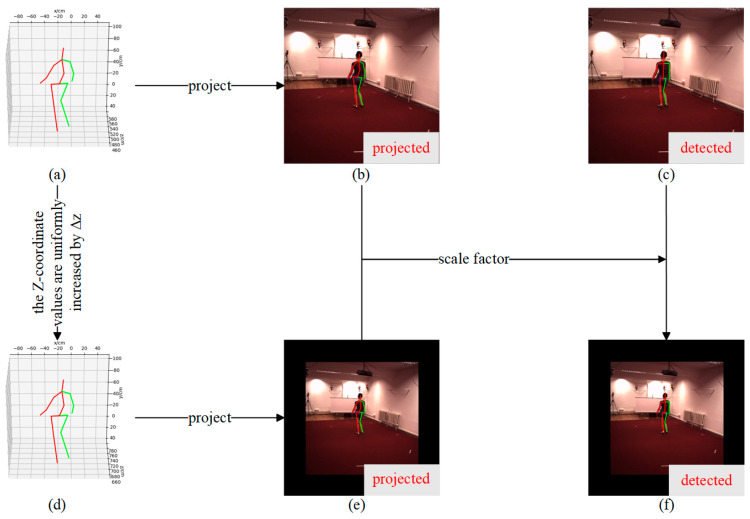
Illustration of depth augmentation (Human3.6M as an example). (**a**) 3D pose in the camera coordinate system before augmentation. (**b**) Projected 2D pose before augmentation. (**c**) 2D pose detected by the 2D pose detector before augmentation. (**d**) Projected 2D pose after augmentation. (**e**) Augmented 3D pose after augmentation. (**f**) Detected 2D pose after augmentation, obtained via inference with the 2D pose detector. The red and green solid lines indicate the left and right sides of the human skeleton, respectively.

The implementation of depth augmentation is illustrated in Figure 4. Specifically, the 3D coordinates, denoted by $\left(x_{before}^{\left(i\right)},y_{before}^{\left(i\right)},z_{before}^{\left(i\right)}\right)$, of all joints in the camera coordinate system are translated by a displacement Δz along the z-axis. After augmentation, the 3D coordinates of each joint in the camera coordinate system, denoted by $\left(x_{aug}^{\left(i\right)},y_{aug}^{\left(i\right)},z_{aug}^{\left(i\right)}\right)$, satisfy:

(15) $$\left[\begin{array}{c} x_{aug}^{\left(i\right)}\\ y_{aug}^{\left(i\right)}\\ z_{aug}^{\left(i\right)} \end{array}\right]=\left[\begin{array}{c} x_{before}^{\left(i\right)}\\ y_{before}^{\left(i\right)}\\ z_{before}^{\left(i\right)} \end{array}\right]+\left[\begin{array}{c} 0\\ 0\\ \Delta z \end{array}\right]$$

where i is the joint index. Accordingly, the projected image coordinates of each joint before and after augmentation can be obtained as:

(16) $$\left[\begin{array}{c} u_{before}^{\left(i\right)}\\ v_{before}^{\left(i\right)}\\ 1 \end{array}\right]=\frac{1}{z_{before}^{\left(i\right)}}K\left[\begin{array}{c} x_{before}^{\left(i\right)}\\ y_{before}^{\left(i\right)}\\ z_{before}^{\left(i\right)} \end{array}\right]$$

(17) $$\left[\begin{array}{c} u_{aug}^{\left(i\right)}\\ v_{aug}^{\left(i\right)}\\ 1 \end{array}\right]=\frac{1}{z_{aug}^{\left(i\right)}}K\left[\begin{array}{c} x_{aug}^{\left(i\right)}\\ y_{aug}^{\left(i\right)}\\ z_{aug}^{\left(i\right)} \end{array}\right]$$

where $\left(u_{before}^{\left(i\right)},v_{before}^{\left(i\right)}\right)$ and $\left(u_{aug}^{\left(i\right)},v_{aug}^{\left(i\right)}\right)$ denote the projected image coordinates of the i-th joint before and after augmentation, respectively, and K represents the camera intrinsic parameters. When the human body is translated by Δz along the z-axis of the camera coordinate system, its image projection undergoes a uniform scaling about the principal point $\left(c_{x},c_{y}\right)$, i.e., the projection of the camera optical center onto the image plane. The principal point $\left(c_{x},c_{y}\right)$ can be obtained from the camera intrinsic matrix K. The scaling factor r is computed from the projected image coordinates of the joints before and after augmentation as:

(18) $$r=\frac{1}{N}\sum_{i=0}^{N-1}\frac{{\left\|\left(u_{aug}^{i},v_{aug}^{i}\right)-\left(c_{x},c_{y}\right)\right\|}_{2}}{{\left\|\left(u_{before}^{i},v_{before}^{i}\right)-\left(c_{x},c_{y}\right)\right\|}_{2}}$$

The 2D pose $\left(s_{aug}^{i},t_{aug}^{i}\right)$ obtained by the 2D pose detector after augmentation is generated by scaling the pre-augmentation 2D pose $\left(s_{before}^{i},t_{before}^{i}\right)$ produced by the detector about $\left(c_{x},c_{y}\right)$ by a factor of r:

(19) $$\left[\begin{array}{c} s_{aug}^{i}\\ t_{aug}^{i} \end{array}\right]=r\left[\begin{array}{c} s_{before}^{i}\\ t_{before}^{i} \end{array}\right]+\left(1-r\right)\left[\begin{array}{c} c_{x}\\ c_{y} \end{array}\right]$$

#### 3.2.2. Angle Augmentation

On land, human body orientations are relatively limited, whereas underwater divers exhibit much more diverse orientations. This discrepancy restricts the applicability of terrestrial 3D human pose datasets to underwater scenarios. To address this limitation, an angle augmentation method is proposed to improve the generalization capability of the augmented terrestrial datasets for underwater applications.

**Figure 5:**
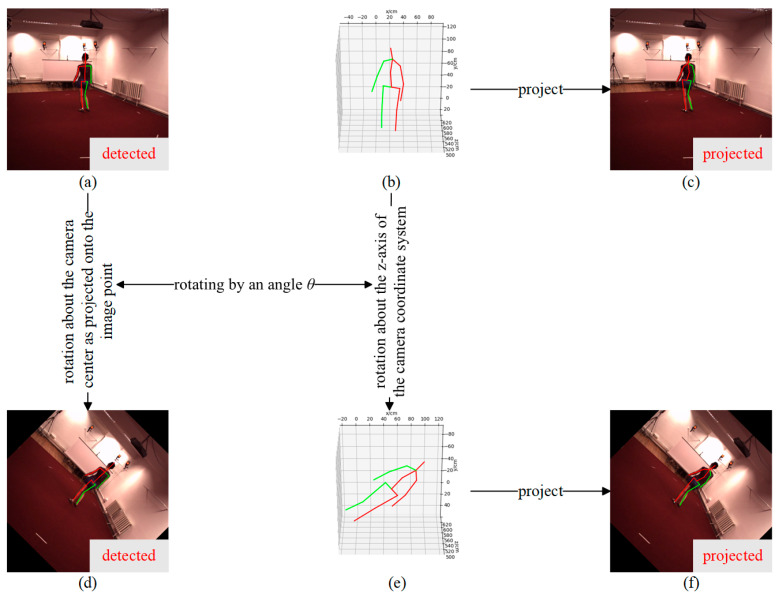
Illustration of view-angle augmentation (Human3.6M as an example). (**a**) 2D pose obtained by the 2D pose detector before augmentation. (**b**) 3D pose in the camera coordinate system before augmentation. (**c**) Projected 2D pose before augmentation. (**d**) 2D pose obtained by the 2D pose detector after augmentation via inference. (**e**) 3D pose in the camera coordinate system after augmentation. (**f**) Projected 2D pose after augmentation. The red and green solid lines indicate the left and right sides of the human skeleton, respectively.

The angle augmentation method is implemented by rotating the human pose about the z-axis of the camera coordinate system by the angle θ, as illustrated in Figure 5. Specifically, the 3D pose $\left(x_{before}^{\left(i\right)},y_{before}^{\left(i\right)},z_{before}^{\left(i\right)}\right)$ of all joints in the camera coordinate system is rotated by θ about the camera z-axis, and the augmented 3D joint coordinates in the camera coordinate system are given by:

(20) $$\left[\begin{array}{c} x_{aug}^{\left(i\right)}\\ y_{aug}^{\left(i\right)}\\ z_{aug}^{\left(i\right)} \end{array}\right]=\left[\begin{array}{ccc} \cos\theta & -\sin\theta & 0\\ \sin\theta & \cos\theta & 0\\ 0 & 0 & 1 \end{array}\right]\left[\begin{array}{c} x_{before}^{\left(i\right)}\\ y_{before}^{\left(i\right)}\\ z_{before}^{\left(i\right)} \end{array}\right]$$

where i is the joint index. The projected image coordinates of each joint before and after augmentation can be computed according to the principles described in Equations (16) and (17). The 2D pose $\left(s_{aug}^{i},t_{aug}^{i}\right)$ obtained by the 2D pose detector after augmentation is generated by rotating the pre-augmentation 2D pose $\left(s_{before}^{i},t_{before}^{i}\right)$ produced by the detector about $\left(c_{x},c_{y}\right)$ by an angle θ:

(21) $$\left[\begin{array}{c} s_{aug}^{i}\\ t_{aug}^{i} \end{array}\right]=\left[\begin{array}{cc} \cos\theta & -\sin\theta \\ \sin\theta & \cos\theta \end{array}\right]\left(\left[\begin{array}{c} s_{before}^{i}\\ t_{before}^{i} \end{array}\right]-\left[\begin{array}{c} c_{x}\\ c_{y} \end{array}\right]\right)+\left[\begin{array}{c} c_{x}\\ c_{y} \end{array}\right]$$

#### 3.2.3. Combined Augmentation

To train the proposed VideoPose5CH model, a combination of the two aforementioned augmentation strategies is applied to the Human3.6M dataset [34].

**Figure 6:**
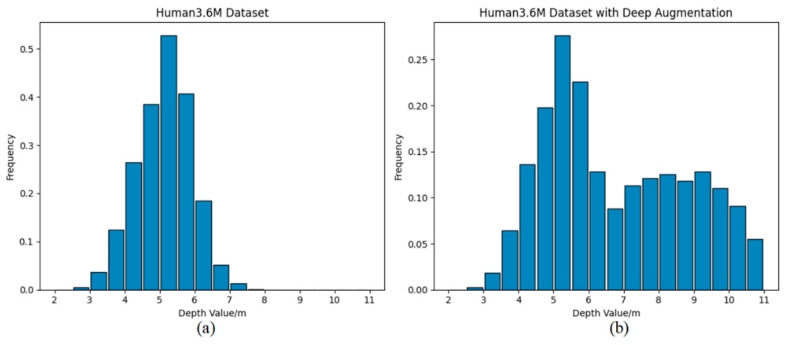
Depth distribution histograms of the Human3.6M dataset and the depth-augmented dataset. The horizontal axis denotes the depth value, and the vertical axis represents the corresponding frequency. (**a**) Depth distribution histogram of all joints across all frames in the Human3.6M dataset; (**b**) Depth distribution histogram of all joints across all frames in the depth-augmented dataset.

First, depth augmentation is performed on the dataset. For each video sequence, the same augmentation depth parameter Δz is applied to all frames, where Δz is randomly selected from a predefined set {2 m,3 m,4 m,5 m}. The depth-augmented data are then merged with the original dataset to form the depth-augmented dataset. Figure 6 illustrates the distribution of the joints’ z-coordinates in the camera coordinate system before and after depth augmentation across all video frames. Before augmentation, the camera-depth values of the joints (i.e., the z-coordinates in the camera coordinate system) are mainly concentrated between 2.5 m and 8 m, whereas after augmentation, the distribution range is expanded to 2.5 m to 11 m. This expansion effectively enhances the generalization ability of VideoPose5CH across different depths.

After completing depth augmentation, angle augmentation is subsequently applied to the resulting dataset. For each action performed by every subject in the Human3.6M dataset, four synchronized multi-view video streams are available, captured by four cameras. During angle augmentation, two of these four videos are randomly selected for angle augmentation, with a rotation angle of $\theta \in \left[\varphi -5{}^{\circ},\varphi +5{}^{\circ}\right],\varphi \in \{n\cdot 30{}^{\circ}:n=0,1,2,\ldots ,11\}$.

Finally, the dataset processed by the two aforementioned augmentation strategies is used as the training dataset for the proposed VideoPose5CH model.

## 4. Experimental Evaluation

In this section, the experimental setup is first described to clarify the evaluation settings. A series of experiments is then conducted to assess the performance of the proposed 6-DoF position and orientation estimation method. Finally, the effectiveness of the proposed dataset augmentation approach is validated through additional experiments.

### 4.1. Experimental Setup

The HRNet-W48 model [10] is used as the 2D pose detector to achieve higher joint localization accuracy, and the input image resolution is set to 256 × 256 pixels. To adapt the model to underwater diver’s pose estimation, 3170 underwater diver images were collected and annotated following the joint definition of the MPII human pose dataset [33]. In addition, this study includes a spine joint. Since the MPII dataset does not provide annotations for this joint, the 2D location of the spine joint is defined as the midpoint between the 2D coordinates of the thorax and hip joints.

The model training is conducted in two stages. First, HRNet-W48 was pre-trained on the MPII dataset. It was then fine-tuned on the underwater dataset via transfer learning [35]. During the pretraining stage, training was performed on seven NVIDIA GeForce RTX 4090 GPUs (NVIDIA Corporation, Santa Clara, CA, USA) using the Adam optimizer implemented in PyTorch (PyTorch Foundation, San Francisco, CA, USA). The dataset was split into training and validation subsets with a ratio of 8:2. The batch size was set to 56, and the learning rate was initialized to 0.00025. The learning rate was decayed by a factor of 0.1 at the 100th and 200th epochs, respectively. The total number of training epochs was 210, and the overall training time was 17 h and 7 min. In the fine-tuning stage on the underwater dataset, four NVIDIA GeForce RTX 4090 GPUs were used, and the Adam optimizer was likewise adopted. The dataset was divided using the same 8:2 ratio. The batch size was set to 16, and the initial learning rate was set to 0.00019. A decay factor of 0.1 was applied to the learning rate at the 150th and 200th epochs. The total number of epochs was 210, and the training process required approximately 3 h and 46 min. After completion of training, the model achieving the lowest validation loss was selected as the final 2D pose detector.

The VideoPose5CH model is trained using the augmented Human3.6M dataset described in Section 3.2.3. Specifically, data from subjects S1, S5, S6, S7, and S8 were used for training, while data from subjects S9 and S11 were reserved for validation. Training was conducted on four NVIDIA GeForce RTX 4090 GPUs using the Adam optimizer. The batch size was set to 8192, and the learning rate was initialized to 0.00038. The model was trained for a total of 230 epochs, with an overall training time of approximately 10 h and 56 min. To achieve the lowest validation loss, the hyperparameters were configured as follows: the hyperparameter β of the Smooth L1 loss (Equation (2)) was set to β=0.2, and the weighting hyperparameter λ in the total loss (Equation (3)) was set to λ=0.46. Finally, the model achieving the lowest overall loss was selected as the inference model.

**Figure 7:**
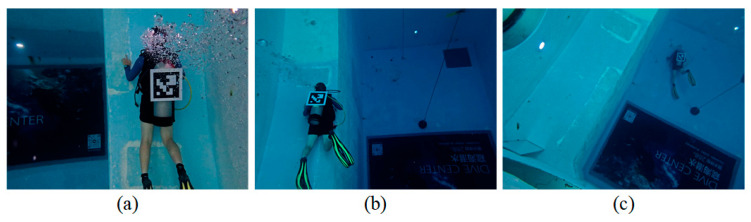
Three videos captured under different camera-to-diver distance conditions are used for the evaluation experiments: (**a**) close-range scenario, (**b**) medium-range scenario, and (**c**) long-range scenario. Note: The text in the background is part of the environment in the diving club, and it is not part of the study.

To evaluate the proposed 6D position and orientation estimation method and to validate the effectiveness of the dataset augmentation strategy, diver videos were collected under three camera-to-diver distance conditions: close-range, medium-range, and long-range scenarios. Across all scenarios, the same diver wearing the same set of diving equipment was recorded. In the close-range scenario, the diver-to-camera distance ranges from 2.643 m to 3.499 m, with the video recorded when the diver was about to move out of the image frame. In the medium-range scenario, the distance ranges from 4.484 m to 6.029 m. In the long-range scenario, the distance ranges from 10.070 m to 11.477 m, corresponding to the diver approaching the pool bottom and the camera just being submerged underwater. Representative examples of these scenarios are shown in Figure 7. To conduct the evaluation, the ground-truth 6-DoF position and orientation of the diver in the videos are required. Therefore, an ArUco marker board of size 35 cm × 35 cm is mounted on the scuba tank, which was rigidly attached to the diver. The marker-board position and orientation were estimated using a PnP algorithm, from which the diver’s ground-truth position and orientation were derived.

### 4.2. Evaluation of 6-DoF Position and Orientation Estimation

#### 4.2.1. Accuracy Analysis of Position and Orientation

This experiment evaluates the accuracy of the proposed diver’s 6-DoF position and orientation estimation algorithm under three scenarios, as illustrated in Figure 7.

To comprehensively assess the performance of the proposed 6-DoF position and orientation estimation method, five evaluation metrics are adopted: (i) component-wise errors for each degree of freedom, (ii) translation error, (iii) rotation error, (iv) relative translation error (RTE), and (v) Average Distance of Model Points (ADD).

Specifically, the component-wise errors correspond to the estimation errors along the three-dimensional translation axes and the three-dimensional rotation axes, thereby reflecting the estimation accuracy for each degree of freedom. The translation error measures the discrepancy between the estimated and ground-truth positions, whereas the rotation error quantifies the discrepancy between the estimated and ground-truth orientations. Their mathematical formulations are given as follows:

(22) $$Error_{trans}={\left\|t-\hat{t}\right\|}_{2}$$

(23) $$Error_{rot}=\arccos\left(\frac{\mathrm{trace}(R{\hat{R}}^{T})-1}{2}\right)$$

where t and $\hat{t}$ denote the ground-truth translation vector and the estimated translation vector, respectively, while R and $\hat{R}$ denote the ground-truth rotation matrix and the estimated rotation matrix, respectively. In addition, the relative translation error (RTE) is further employed to normalize the translation error, to measure its ratio with respect to the magnitude of the ground-truth translation vector. The mathematical definition is given by:

(24) $$\mathrm{RTE}=\frac{{\left\|t-\hat{t}\right\|}_{2}}{{\left\|t\right\|}_{2}}$$

The ADD metric measures the mean 3D distance between corresponding keypoints transformed by the estimated and the ground-truth position and orientation, respectively. As a criterion that jointly accounts for both translation and rotation errors, its mathematical definition is given by:

(25) ADD=1M∑PDiver∈MRPDiver+t−R^PDiver+t^2

where PDiver denotes a joint in the joints set M. In the study, ADD(0.5) denotes the proportion of samples whose ADD error is below a threshold set to 0.5 times the subject’s body height.

**Figure 8:**
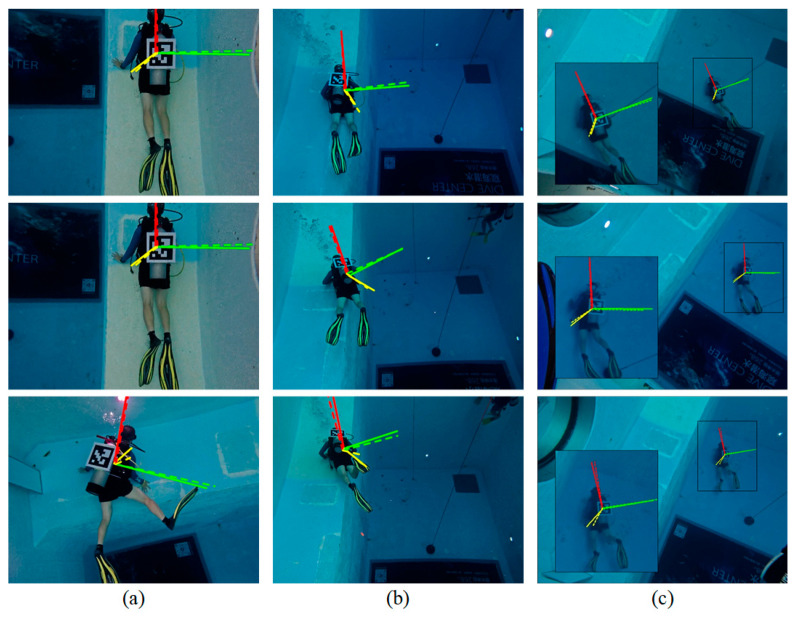
Diver’s 6-DoF position and orientation estimation results in the three scenarios. The position and orientation are visualized as the axes of a Cartesian coordinate frame, where the red, green, and yellow axes correspond to the x-, y-, and z-axis directions, respectively. The ground-truth position and orientation are shown with solid axes, while the estimated position and orientation are shown with dashed axes. The three columns (**a**–**c**) correspond to the three scenarios. Overall, the estimated coordinate frames show good agreement with the ground truth across all scenarios.

The position and orientation estimation results of the proposed algorithm across three scenarios are presented in Figure 8. The estimated diver’s position and orientation are visualized by a dashed tri-axial Cartesian coordinate frame, while the ground-truth results are indicated by a solid coordinate frame. The results demonstrate that the proposed method can reliably estimate the diver’s 6-DoF position and orientation over a wide range of distances (2.643–11.477 m).

**Figure 9:**
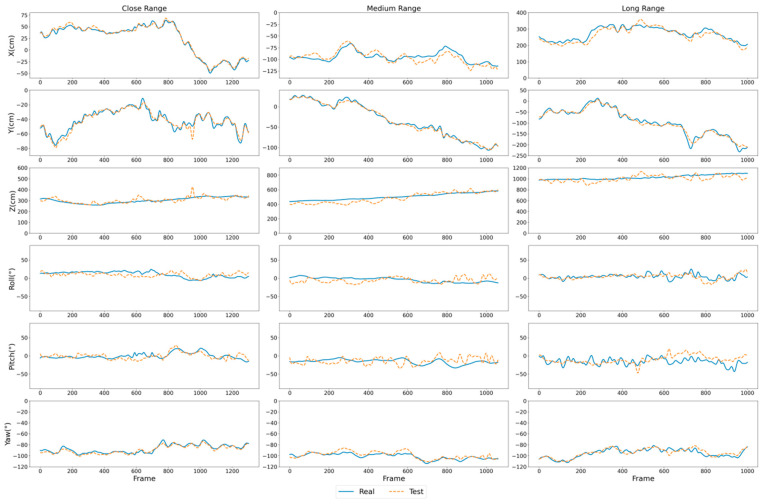
Comparison curves between the estimated and ground-truth 6-DoF position and orientation in three scenarios. The blue curves indicate the ground truth, and the orange curves indicate the estimated results. The X, Y, Z, and yaw components exhibit close tracking between estimation and ground truth across all scenarios, while relatively larger deviations are observed in the roll and pitch components.

In Figure 9, the estimated and ground-truth curves of the diver’s position and orientation components are presented and compared for three scenarios. It can be seen that the estimated curves of the X, Y, Z and yaw components match the ground-truth curves well. In particular, in the far-range scenario, a high level of agreement is maintained even at distances from 10.070 m to 11.477 m. By contrast, the estimated roll and pitch components deviate more noticeably from the ground truth. This is mainly because the ArUco marker board attached to the diver is relatively large; during motion, the scouring effect of the water flow may cause slight changes in the relative orientation between the marker board and the diver. Nonetheless, the estimated roll and pitch curves still exhibit variations around the corresponding ground-truth curves, suggesting that the proposed method remains effective in estimating the roll and pitch components.

The error statistics of the proposed method for diver’s 6-DoF position and orientation estimation under different scene conditions are summarized in Table 2, including component-wise errors for each degree of freedom, translation error, rotation error, RTE, and the ADD metric. The results indicate that, in the close-range scenario, the proposed method achieves high accuracy. The errors of the three translation components remain low: the errors in the X and Y directions are both below 3 cm, while the error in the Z direction is 12.77 cm. In addition, the overall translation error and RTE are 13.84 cm and 4.46%, respectively. For rotation, the errors in roll, pitch, and yaw are 7.32°, 6.14°, and 2.14°, respectively, with an overall orientation error of 10.84°. In this scenario, ADD(0.5) reaches 99.23%, indicating that the proposed method provides accurate and stable estimation performance at close range.

As the distance increases, errors generally rise. In the medium-range scenario, the translation error along the Z axis increases to 29.85 cm, with an overall translation error of 31.48 cm and an RTE of 6.33%. The rotation errors also increase slightly across the degrees of freedom, yielding an overall orientation error of 14.44°. Meanwhile, ADD(0.5) remains at 96.98%, suggesting that the method retains strong robustness at medium range.

In the long-range scenario, due to the reduced target scale and the degradation of underwater imaging, the translation error further increases, particularly in depth. The error in the Z direction reaches 43.01 cm, and the overall translation error is 47.37 cm. Although the absolute error is relatively large, considering that the camera-to-target distance ranges from 10.070 m to 11.477 m, the relative translation error is 4.41%, which still corresponds to a high level of accuracy. The rotation errors remain comparatively stable, with an overall orientation error of 12.86°. Although ADD(0.5) decreases to 80.92%, the proposed method still maintains acceptable position and orientation estimation performance at long range.

Overall, across the three scenarios, the proposed method achieves an average translation error of 29.38 cm, an average orientation error of 12.58°, an average RTE of 5.04%, and a mean ADD(0.5) of 93.07% over the entire test set. These results demonstrate that the proposed method can provide stable and reliable diver’s 6-DoF position and orientation estimation under varying observation distances. In addition, experiments conducted on a computer equipped with an NVIDIA GeForce RTX 3060 GPU (6 GB VRAM; NVIDIA Corporation, Santa Clara, CA, USA), an AMD Ryzen 7 5800H CPU (Advanced Micro Devices, Inc., Santa Clara, CA, USA), and 16 GB RAM (Samsung Electronics Co., Ltd., Suwon, Republic of Korea) demonstrate that the proposed method achieves an inference speed of 18.2 FPS.

#### 4.2.2. Comparative Analysis

At present, publicly available studies on monocular vision–based 6-DoF human position and orientation estimation remain limited. To systematically evaluate the localization accuracy of the proposed method, PULP-Frontnet [36], one of the most representative and informative works in this area, is selected as the primary comparison baseline. PULP-Frontnet is a direct regression–based method for estimating position and orientation. A systematic evaluation of our method in terms of position and orientation estimation accuracy is then conducted.

The PULP-Frontnet model was trained on the Human3.6M dataset augmented using the method described in Section 3.2.3. During training, the images in the augmented data were obtained by applying the same 2D-detector-based pose augmentation to the original images, while the corresponding labels were computed from the 3D human pose in the camera coordinate system. Since PULP-Frontnet outputs only a single-axis orientation angle (rotation about the gravity axis), which corresponds to the diver’s pitch relative to the camera, PULP-Frontnet is first used to predict the diver’s pitch in the camera coordinate system. This prediction is then compared with the estimate produced by our method.

**Figure 10:**
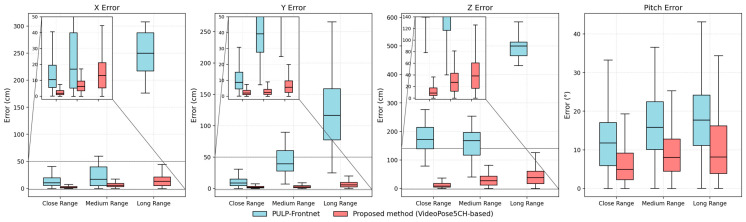
Box plots of the errors in 4-DoF for the two methods across the three scenarios. The proposed method generally shows lower errors and narrower distributions than PULP-Frontnet across the three scenarios.

The box plots of the errors of the two methods for four degrees of freedom (X, Y, Z, and pitch) across the three scenarios are shown in Figure 10, and Table 3 lists the overall mean errors of position and orientation estimation for both methods in each scenario. The results indicate that, for position estimation, the proposed method yields substantially smaller errors than PULP-Frontnet along all three axes (X, Y, and Z), reducing both the overall translation error and the relative translation error by approximately one order of magnitude. For orientation estimation, although PULP-Frontnet focuses on single-axis angle prediction, our method still achieves higher accuracy in pitch estimation, demonstrating strong robustness and accuracy in orientation solving as well.

In addition, to further analyze the performance differences between the two methods under comparisons of different single-axis orientation angles, PULP-Frontnet is also used to predict the diver’s roll and yaw angles with respect to the camera coordinate system, and these predictions are compared with the corresponding estimates obtained by our method. The results are reported in Table 4 and Table 5, respectively. It can be observed that, when PULP-Frontnet is used to estimate roll or yaw, its angular errors increase remarkably, and the accuracy gap relative to our method becomes substantially larger than that observed for pitch estimation. In contrast, our method maintains high accuracy and stability for both roll and yaw estimation.

The comparative results demonstrate that our method not only provides a clear advantage in position estimation but also exhibits stronger generalization capability and higher overall accuracy in three-axis orientation-angle estimation, thereby validating its effectiveness and superiority for diver’s 6-DoF position and orientation estimation.

### 4.3. Ablation Study

To evaluate the impact of the refined 2D pose output by VideoPose5CH on the performance of the diver’s 6-DoF position and orientation estimation, the 2D joint coordinates used by the PnP solver are replaced with the current-frame 2D joint coordinates produced by the 2D pose detector, rather than the refined 2D coordinates output by VideoPose5CH. Under this setting, the 2D refinement capability of VideoPose5CH is not utilized. For a fair comparison, VideoPose5CH is further replaced with the VideoPose3D model [12]. VideoPose3D is trained on the augmented Human3.6M dataset described in Section 3.2.3, and the model with the lowest validation loss is selected for inference. For clarity, this baseline is referred to as HRNet + VideoPose3D, while the original method is denoted as HRNets + VideoPose5CH.

The experimental results are summarized in Table 6 and Table 7. Table 6 compares the translation error, rotation error, RTE, and the ADD(0.5) metric of the two methods across the three scenarios. It can be observed that HRNets + VideoPose5CH consistently outperforms HRNet + VideoPose3D, which does not employ 2D pose refinement, in all scenarios and on all evaluation metrics, with a particularly pronounced performance gap in the close-range scenario. Table 7 further reports the overall mean statistics for the three scenarios, including the mean errors of each 6-DoF component, the mean translation error, the mean rotation error, and the mean RTE. The results in Table 7 indicate that HRNets + VideoPose5CH achieves superior performance across all metrics.

The higher accuracy of HRNets + VideoPose5CH compared with HRNet + VideoPose3D is primarily attributed to the fact that the 2D pose detector and the 3D pose estimator (i.e., VideoPose5CH or VideoPose3D) are trained on two different datasets, where the joint definitions cannot be guaranteed to be fully consistent. Specifically, for some joints, the same joint name may refer to slightly different anatomical landmarks across the two datasets. As a result, in HRNet + VideoPose3D, the 2D and 3D keypoints provided to the PnP solver are not perfectly matched, leading to larger estimation errors. In contrast, in HRNets + VideoPose5CH, the 2D refinement mechanism of VideoPose5CH enforces consistency between the 2D and 3D joints used as PnP inputs, thereby yielding more accurate position and orientation estimations. This effect is most remarkable in the close-range scenario: because the diver occupies a larger portion of the image, the pixel-level discrepancies caused by inconsistent joint definitions in HRNet + VideoPose3D are further amplified, resulting in a larger performance gap between the two methods. Overall, this ablation study demonstrates that the 2D refinement capability of VideoPose5CH plays a crucial role in improving the robustness of 6-DoF position and orientation estimation.

### 4.4. Evaluation of the Effectiveness of the Dataset Augmentation Method

This experiment aims to evaluate the effectiveness of the proposed dataset augmentation method. Therefore, VideoPose5CH is trained using three training sets: the original Human3.6M dataset without augmentations (denoted as “no depth, no angle”), the dataset augmented with depth augmentation only (denoted as “depth, no angle”), and the dataset jointly augmented as described in Section 3.2.3 (denoted as “depth, angle”). By comparing the diver’s position and orientation estimation accuracy of models trained on these three datasets across the three scenarios, the impact of the proposed augmentation strategy is assessed. In this experiment, only the training dataset for VideoPose5CH is changed, while all other settings in the diver’s 6-DoF position and orientation estimation framework remain unchanged.

**Figure 11:**
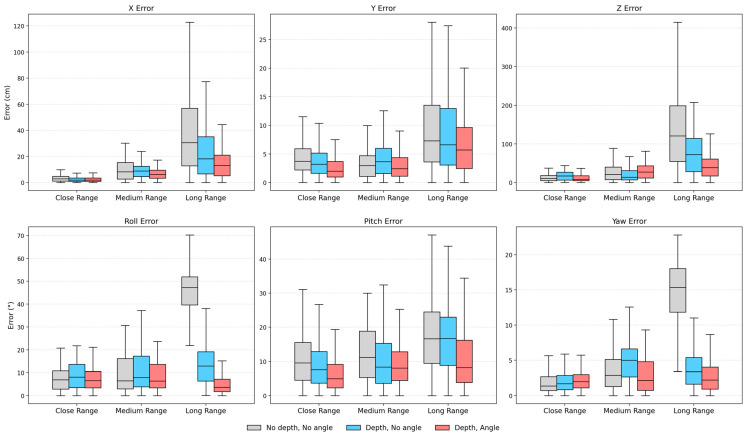
Box plots comparing the 6-DoF position and orientation estimation errors across the three scenarios under different training datasets. Overall, as depth and angle augmentations are progressively applied, most error components exhibit a decreasing tendency and more concentrated distributions, although slight fluctuations are observed in certain components. The most evident improvements occur in the long-range scenario.

The experimental results are summarized in Figure 11, as well as in Table 8 and Table 9. Figure 11 presents a comparison of the box plots for 6-DoF position and orientation estimation errors across close-range, medium-range, and long-range scenarios under different training-dataset configurations. Overall, both depth augmentation and angle augmentation are shown to effectively reduce the component-wise errors and, to some extent, yield more concentrated error distributions. For the close-range and medium-range scenarios, the improvements brought by augmentations are relatively limited. In the close-range scenario, the errors of the Y component and the pitch component decrease consistently as the two augmentation strategies are applied, whereas a slight increase is observed in the yaw error; nevertheless, this increase remains smaller than the corresponding reduction in the pitch error. In the medium-range scenario, compared with training without augmentation, the combined effect of the two augmentations is a reduction in five out of the 6-DoF component errors, except for the Z component. In contrast, the most pronounced improvements are observed in the long-range scenario: as depth augmentation and angle augmentation are progressively applied, the component-wise errors exhibit an overall stepwise decrease (except that the pitch component changes only marginally between the datasets before and after depth augmentation), and the error distributions become noticeably more concentrated. A further comparison of the two augmentation strategies reveals that depth augmentation achieves a larger overall error reduction than angle augmentation, with particularly notable improvements in the Z, roll, and yaw components.

In Table 8 and Table 9, a further quantitative summary of the translation error, rotation error, RTE, and the ADD(0.5) metric is provided for each of the three scenarios and for the overall average under different training-dataset configurations. The results show that, in terms of translation and rotation errors, the combined depth augmentation and angle augmentation configuration achieves the lowest errors in three scenarios except for the translation error in the medium-range scenario, where using depth augmentation yields the best performance. The RTE exhibits a trend consistent with that of the translation error. For the ADD(0.5) metric, the close-range scenario exhibits only minor differences before and after augmentations, whereas both the medium-range and long-range scenarios show an increasing trend as the two augmentation strategies are introduced, indicating a sustained improvement in position and orientation estimation accuracy. Considering the overall averaged results, progressively incorporating the augmentation strategies leads to an overall reduction in translation and rotation errors and an overall increase in ADD(0.5).

In summary, the proposed dataset augmentation method effectively improves the accuracy of diver’s 6-DoF position and orientation estimation and enhances the robustness and applicability of the proposed approach across different distance scenarios.

## 5. Conclusions

To address the challenge of diver–robot collaborative position and orientation perception in underwater environments, this study proposes a method for estimating the diver’s 6-DoF position and orientation, which is built upon a novel network named VideoPose5CH. In the proposed approach, VideoPose5CH is employed to estimate the diver’s 3D human pose while simultaneously producing a refined 2D human pose. Given the scarcity of underwater 3D human pose datasets, a terrestrial 3D human pose dataset augmentation method tailored to underwater scenarios is further developed, targeting 3D human pose estimation approaches based on 2D-to-3D lifting. Experimental results show that the proposed method achieves high accuracy in 6-DoF position and orientation estimation and also confirm the effectiveness of the adopted dataset augmentation strategy.

Further research is still required. First, it is necessary to enhance the adaptability of 6-DoF localization algorithms to complex underwater environments. In such environments, underwater imaging is severely affected by optical phenomena, including light absorption, scattering, color distortion, and blurring. In future work, image enhancement techniques will be incorporated as front-end components to improve the environmental robustness of 6-DoF position and orientation estimation algorithms. Second, the proposed dataset augmentation method is mainly limited to geometric transformations of human poses and does not yet incorporate image-level augmentation strategies for underwater scenes. Considering that real-world underwater images are affected by color distortion and image degradation, future research will explore more realistic underwater data generation approaches tailored to diver scenarios, to simultaneously obtain high-quality underwater images as well as accurate 3D human pose and 6-DoF position and orientation annotations. Third, to facilitate the practical deployment of the proposed algorithm on AUVs, its real-time performance should be further improved. In addition, to further improve the overall performance of 6-DoF position and orientation estimation, the estimation accuracy should be continuously refined. Finally, although the proposed method has achieved promising experimental results on existing datasets, its generalization capability under varying diver body morphologies and diverse diving equipment configurations has yet to be thoroughly validated. Therefore, future work will focus on constructing a more diverse diver dataset and conducting systematic validation experiments under varying body morphologies and diverse diving equipment configurations, in order to further assess and enhance the model’s adaptability and robustness in complex underwater environments.

## Figures and Tables

**Table 1:** Coordinate notation for selected human joints in the root-joint coordinate system.

Joint Name	Coordinate Representation in the Root-Joint Coordinate System
Hip	PHipRoot
Spine	PSpineRoot
Thorax	PThoraxRoot
Left shoulder	PLShoulderRoot
Right shoulder	PRShoulderRoot

**Table 2:** Error results of driver 6-DoF position and orientation estimation.

Scenario	*X* Error (cm)	*Y* Error (cm)	*Z* Error (cm)	Roll Error (°)	Pitch Error (°)	Yaw Error (°)	Trans Err (cm)	RTE	Orient Err (°)	ADD (0.5)
Close Range	2.43	2.62	12.77	7.32	6.14	2.14	13.84	4.46%	10.84	99.23%
Medium Range	6.61	2.89	29.85	8.13	9.37	3.03	31.48	6.33%	14.44	96.98%
Long Range	14.47	7.10	43.01	5.27	10.53	2.82	47.37	4.41%	12.86	80.92%
Overall Mean	7.33	4.04	27.15	6.96	8.47	2.62	29.38	5.04%	12.58	93.07%

**Table 3:** Comparison of the overall mean position and orientation estimation error between the two methods, with the single-axis attitude angle in PULP-Frontnet set to the pitch angle.

Method	*X* Error (cm)	*Y* Error (cm)	*Z* Error (cm)	Pitch Error (°)	Trans Err (cm)	RTE
PULP-Frontnet [36]	86.54	54.80	265.13	15.42	293.00	48.98%
Proposed method (VideoPose5CH-based)	**7.33**	**4.04**	**27.15**	**8.47**	**29.38**	**5.04%**

Bold font indicates the optimal value.

**Table 4:** Comparison of the overall mean position and orientation estimation error between the two methods, with the single-axis attitude angle in PULP-Frontnet set to the roll angle.

Method	*X* Error (cm)	*Y* Error (cm)	*Z* Error (cm)	Roll Error (°)	Trans Err (cm)	RTE
PULP-Frontnet [36]	94.10	65.93	272.57	48.98	303.48	52.90%
Proposed method (VideoPose5CH-based)	**7.33**	**4.04**	**27.15**	**6.96**	**29.38**	**5.04%**

Bold font indicates the optimal value.

**Table 5:** Comparison of the overall mean position and orientation estimation error between the two methods, with the single-axis attitude angle in PULP-Frontnet set to the yaw angle.

Method	*X* Error (cm)	*Y* Error (cm)	*Z* Error (cm)	Yaw Error (°)	Trans Err (cm)	RTE
PULP-Frontnet [36]	86.49	61.30	276.15	88.53	303.47	50.28%
Proposed method (VideoPose5CH-based)	**7.33**	**4.04**	**27.15**	**2.62**	**29.38**	**5.04%**

Bold font indicates the optimal value.

**Table 6:** Comparison of translation error, rotation error, and ADD(0.5) between the two methods under the three scenarios in the ablation study.

Method	Close Range				Medium Range				Long Range			
	Trans Err (cm)	RTE	Orient Err (°)	ADD (0.5)	Trans Err (cm)	RTE	Orient Err (°)	ADD (0.5)	Trans Err (cm)	RTE	Orient Err (°)	ADD (0.5)
HRNet + VideoPose3D	93.18	31.11%	32.80	52.72%	41.37	8.17%	24.00	86.89%	61.71	5.90%	15.05	68.03%
HRNets + VideoPose5CH	**13.84**	**4.46%**	**10.84**	**99.23%**	**31.48**	**6.33%**	**14.44**	**96.98%**	**47.37**	**4.41%**	**12.86**	**80.92%**

Bold font indicates the optimal value.

**Table 7:** Comparison of the overall mean 6-DoF error, as well as the mean translation and rotation errors aggregated over the three scenarios in the ablation study.

Method	*X* Error (cm)	*Y* Error (cm)	*Z* Error (cm)	Roll Error (°)	Pitch Error (°)	Yaw Error (°)	Trans Err (cm)	RTE	Orient Err (°)
HRNet + VideoPose3D	11.55	7.24	64.95	11.27	12.33	10.63	67.49	16.38%	24.75
HRNets + VideoPose5CH	**7.33**	**4.04**	**27.15**	**6.96**	**8.47**	**2.62**	**29.38**	**5.04%**	**12.58**

Bold font indicates the optimal value.

**Table 8:** Comparison of translation error, rotation error, RTE, and ADD for 6-DoF position and orientation estimation under three scenarios, using different training datasets.

Datasets	Close Range				Medium Range				Long Range			
	Trans Err (cm)	RTE	Orient Err (°)	ADD (0.5 h)	Trans Err (cm)	RTE	Orient Err (°)	ADD (0.5 h)	Trans Err (cm)	RTE	Orient Err (°)	ADD (0.5 h)
No depth, no angle	15.02	4.9%	14.13	**100.0%**	29.79	5.64%	17.63	94.81%	151.02	14.35%	50.73	31.17%
Depth, no angle	18.58	6.16%	14.47	**100.0%**	**26.65**	**5.11%**	21.43	96.04%	81.26	7.67%	23.62	49.45%
Depth, angle	**13.84**	**4.46%**	**10.84**	99.23%	31.48	6.33%	**14.44**	**96.98%**	**47.37**	**4.41%**	**12.86**	**80.92%**

Bold font indicates the optimal value.

**Table 9:** Comparison of the overall mean translation error, mean rotation error, mean RTE, and mean ADD for 6-DoF position and orientation estimation under three scenarios, using different training datasets.

Datasets	Overall Mean			
	Trans Err (cm)	RTE	Orient Err (°)	ADD (0.5 h)
No depth, no angle	60.14	7.95%	26.13	77.88%
Depth, no angle	39.77	6.28%	19.38	83.71%
Depth, angle	**29.38**	**5.04%**	**12.58**	**93.07%**

Bold font indicates the optimal value.

## Data Availability

Access to the data will be considered upon request by the authors.

## Abbreviations

The following abbreviations are used in this manuscript:

6-DoF	six-degree-of-freedom
ADD	Average Distance of Model Points
CNN	Convolutional Neural Network
CPM	Convolutional Pose Machines
CPN	Cascaded Pyramid Network
DNN	Deep Neural Network
ELA	Efficient Local Attention
HRNet	High-Resolution Network
IEF	Iterative Error Feedback
KNN	k-nearest neighbors
LiDAR	light detection and ranging
LSTM	Long Short-Term Memory network
MoCap	motion capture
MPJPE	mean per-joint position error
PAF	Part Affinity Field
PnP	Perspective-n-Point
RANSAC	Random Sample Consensus
RTE	relative translation error
AUV	Autonomous Underwater Vehicle
